# News and views

**DOI:** 10.1007/s43673-021-00019-y

**Published:** 2021-08-17

**Authors:** AAPPS

**Affiliations:** Association of Asia Pacific Physical Societies, Pohang, South Korea

## Nagoya University Accelerator-based Neutron Source (NUANS)

### Introduction

Boron neutron capture therapy (BNCT) is the most attention radiotherapy that can individually destroy cancer cells based on the ^10^B(n, a)^7^Li reaction between boron and neutrons. Research reactors were previously used as a neutron source for BNCT, but most research reactors have already closed or are in the process of shutting down. Accordingly, in lieu of research reactors, several accelerator-driven neutron sources have been constructed or are under construction worldwide.

An accelerator-based neutron source that combines a high-power accelerator and a lithium (Li) target is very advantageous for BNCT from a neutronic point of view. At Nagoya University, we have been developing a sealed Li target with a unique structure and have conducted an accelerator-based neutron BNCT project.

### Nagoya University Accelerator-based Neutron Source (NUANS)

#### Accelerator

**Fig. 1:**
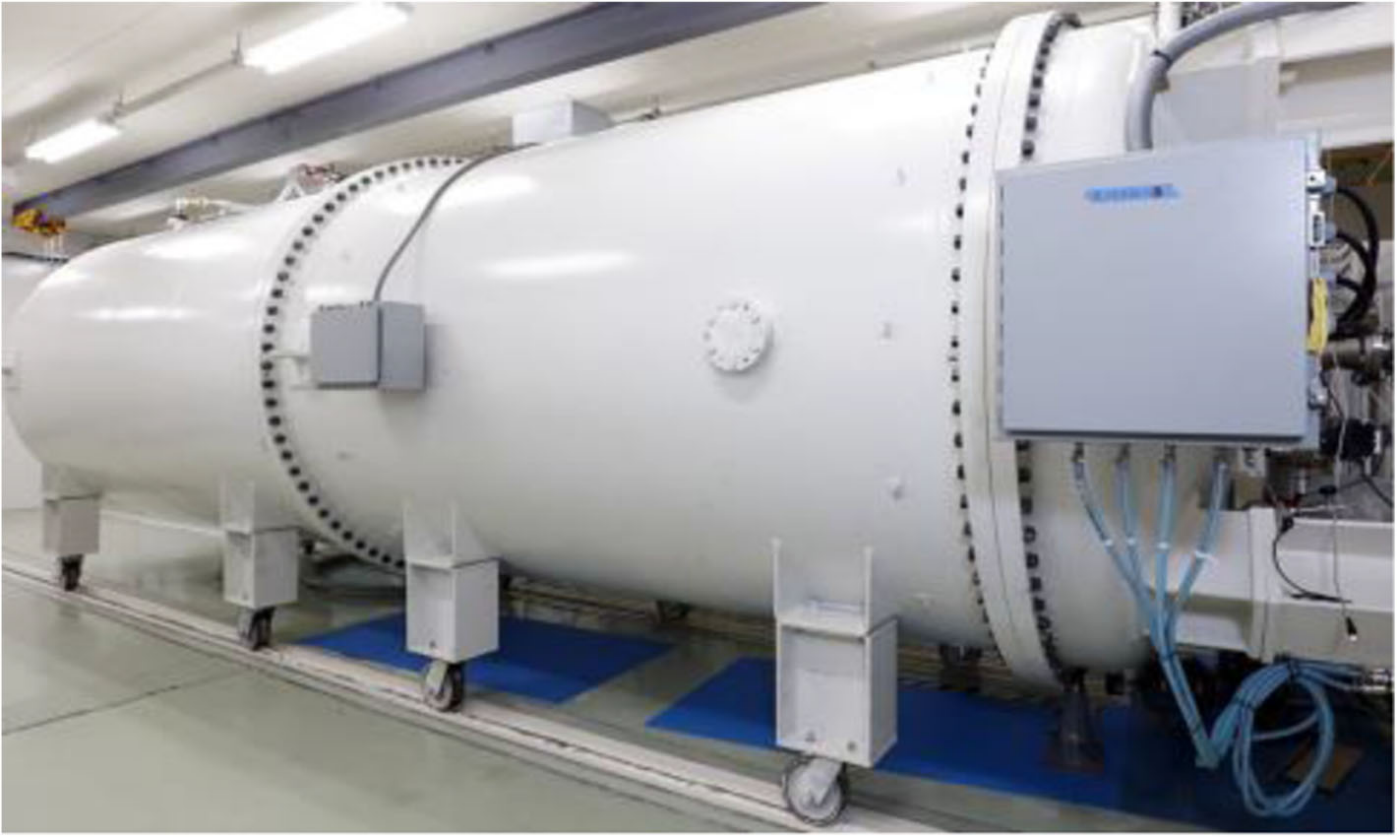
Proton-Dynamitron

In 2015, an electrostatic DC accelerator (Proton-Dynamitron, Ion Beam Applications SA) with proton energy of 2.8 MeV and a maximum current of 15 mA was installed at Nagoya University. Figure 1 shows the appearance of the accelerator. We assembled the beamline, started beam conditioning, and completed the HV conditioning (3.1MV) in 2017.

The proton beam trajectory is controlled by three quadrupole magnets and a set of steering magnets. To estimate the spatial variation of the beam size over the beamline, beam transport analysis was performed by a linear optics model (the 6 by 6 thick lens quadrupole model). In addition, as a beam scanning system was introduced to reduce the heat load on the target, we succeeded in expanding the beam irradiation area. We were able to transport a high-current beam of 8 mA by using beam analysis and beam scanning in 2020.

#### Sealed lithium target

**Fig. 2:**
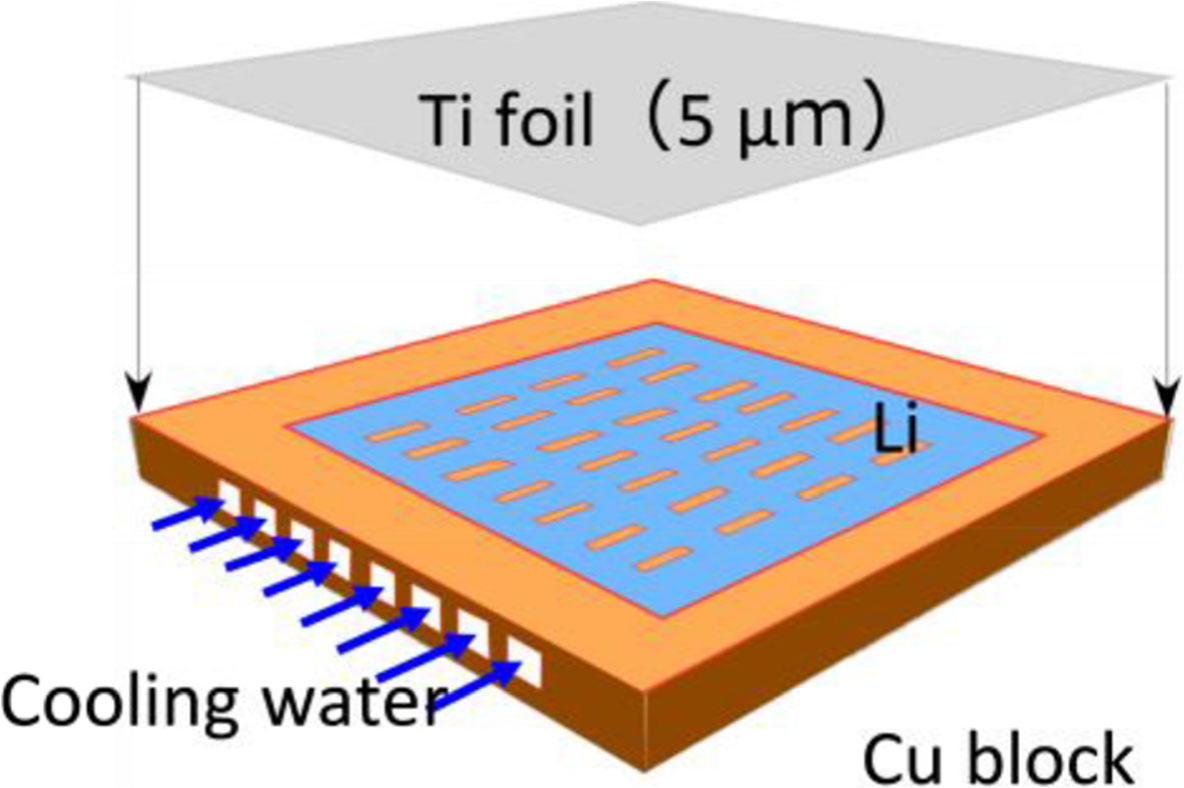
Sealed Li target

A unique developed sealed lithium (Li) target shown in Fig. 2 has a structure where Li metal settles on a cooling plate covered with titanium (Ti) foil. High heat removal efficiency could be achieved by inducing turbulent flow by developing a ribbed cooling water channel [1]. The basic design and production of the sealed Li target structure have been completed, and an endurance test by proton beam irradiation is currently being conducted. No damage was observed on the Ti foil after proton beam irradiation with a beam power density of 5.7 MW/m^2^ for a total of 50 h.

#### Beam shaping assembly

The beam shaping assembly (BSA) of NUANS has a unique system with a nozzle for a compact gantry system. Previous designs and preliminary experiments have shown that the neutron field emitted from the nozzle conforms to International Atomic Energy Agency Technical Documents (IAEA-TECDOC-1223) [2, 3]. Improvement in the BSA and nozzle led to more therapeutically suitable neutron generation.

#### Neutron characteristics

The spatial distribution of thermal neutron in the water phantom was measured by the gold foil activation method. The current of the proton beam used in the experiment was 4mA. The thermal neutron flux was evaluated to be 2.5 × 10^8^ n/cm^2^/s at a distance of about 20 mm from the beam incident surface of the water phantom.

### In Vitro experiments

As the first step to evaluate the performance of NUANS, “in vitro” tests had been performed by using human squamous cancer (SAS) cells. SAS cells were soaked in a medium of 200 ppm boron-phenylalanine (BPA) for over 24 h before the neutron irradiation. The neutron flux had a comparatively flat distribution in the radial direction of the BSA extraction port (12 cm in diameter) and nine 0.6-mL tubes could be set in the irradiation area. Each tube contained a cell suspension of 5 × 10^4^ cells/0.5 mL and was set in an acrylic case dipped on a water phantom for neutron irradiation.

**Fig. 3:**
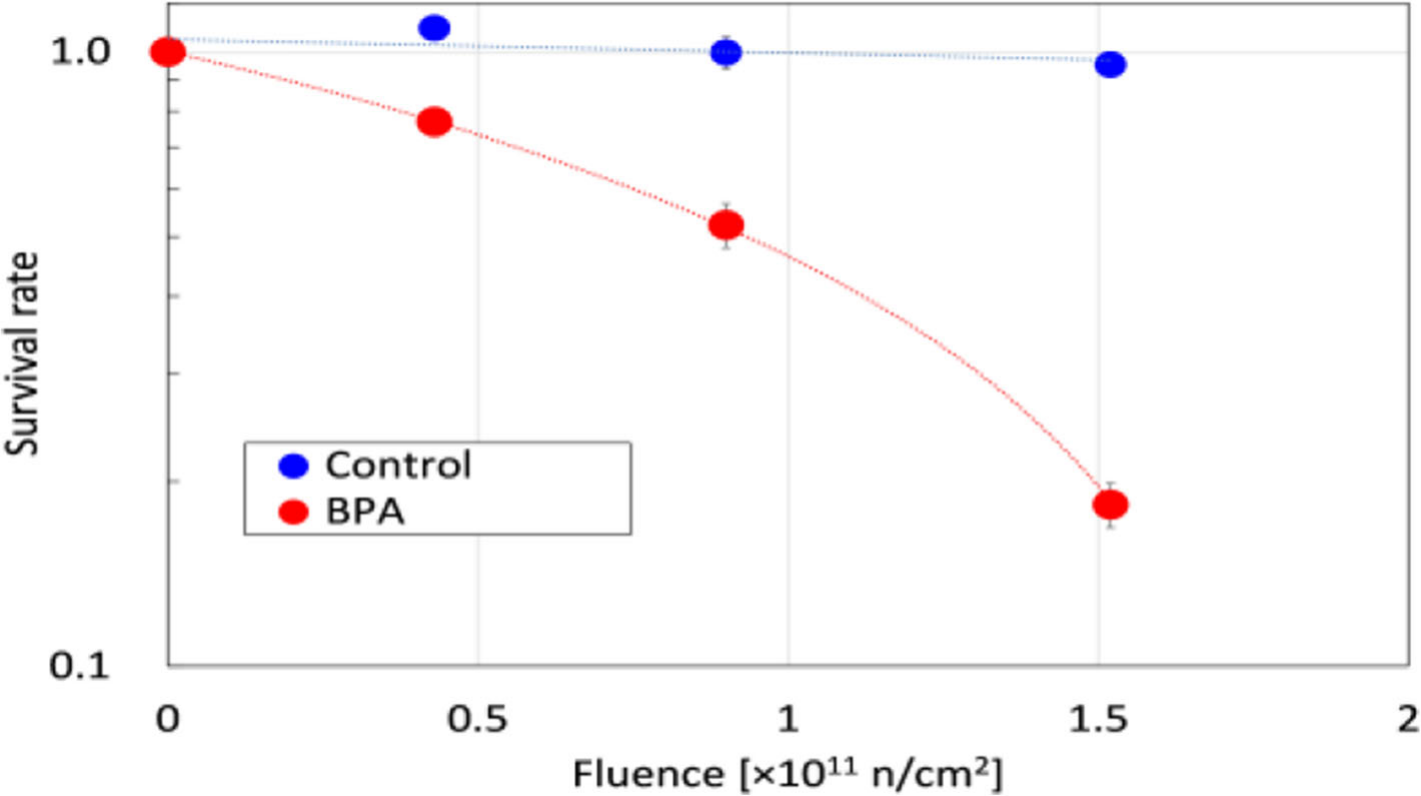
Comparison between the control group and the BPA group

The neutron flux was almost constant during irradiation. The thermal neutron flux was measured in all batches to be about 2.6 × 10^7^ n/s/cm^2^ at the phantom current of 0.5 mA. The total dose was controlled by changing the neutron irradiation time from 10 to 70 min. The gamma-ray dose measured by ionization was 0.3 Gy/h. Figure 3 shows the comparison between the control group without BPA and the BPA group. The BPA group showed neutron fluence-dependent cancer cell damage, while the survival rate of the control group was approximately 1.0 without regard to increase neutron fluence. This suggests that NUANS is a device suitable for BNCT that has a known gamma-ray contamination rate.

### Conclusion

We are working on adjusting the proton beam current to 15 mA and improving the sealed Li target to obtain an epi-thermal neutron flux of 1 × 10^9^ n/cm^2^/s. We are also preparing to conduct in vivo experiments in the near future.

## Development of room-temperature semiconductor gamma-ray detectors with very high detection efficiency

### Abstract

In this study, room-temperature semiconductor gamma-ray detectors with very high detection efficiency were developed from a compound semiconductor, thallium bromide (TlBr). A 1-cm-thick TlBr detector was fabricated from a TlBr crystal grown by the traveling molten zone method using a zone-purified material. The TlBr gamma-ray detector fabricated from the crystal exhibited high energy resolutions at room temperature, reflecting the high charge transport properties of the material. The TlBr detectors are attractive for applications in gamma-ray spectroscopy and imaging.

#### Introduction

A gamma-ray is one of the most penetrative types of ionizing radiation. The applications of gamma-rays are found in various fields, including nuclear medicine, astronomy, in physics experiments, and in nuclear power plants. In order to detect gamma-rays efficiently, high atomic numbers and high density are required for the detector materials. Germanium (Ge) semiconductor detectors are commonly used for detecting gamma-rays with high energy resolutions. However, cryogenic cooling is required for operating Ge detectors by reducing the dark current noise originating from the low bandgap energy of Ge (0.67 eV). Although wide bandgap compound semiconductors such as cadmium telluride and cadmium zinc telluride have been studied and are commercially available as room-temperature semiconductor detectors, more efficient room-temperature gamma-ray detectors are required for nuclear sciences and medical imaging applications. We have studied a compound semiconductor, thallium bromide (TlBr), as a gamma-ray detector material with very high detection efficiency and the capability for room-temperature operation.

#### Thallium bromide

TlBr is a compound semiconductor with a wide bandgap energy of 2.68 eV; this wide bandgap energy allows TlBr detectors to operate at room temperature. TlBr detectors exhibit high detection efficiency for gamma-rays because of their high atomic numbers (81 and 35) and their high density (7.56 g/cm^3^). Although TlBr has been studied as a semiconductor detector material [4], practical use of the detectors was limited, mainly due to the low charge transport properties and the instability of the detectors’ performance.

We have succeeded in improving charge transport properties in TlBr crystals significantly by purifying the starting material by the zone melting method [5]. We found that improvement of the long-term stability of TlBr detectors was realized by application of thallium electrodes to the devices, which suppressed space charge accumulations under the electrodes caused by ionic conductions in the crystals [6].

#### Detector performance

**Fig. 4:**
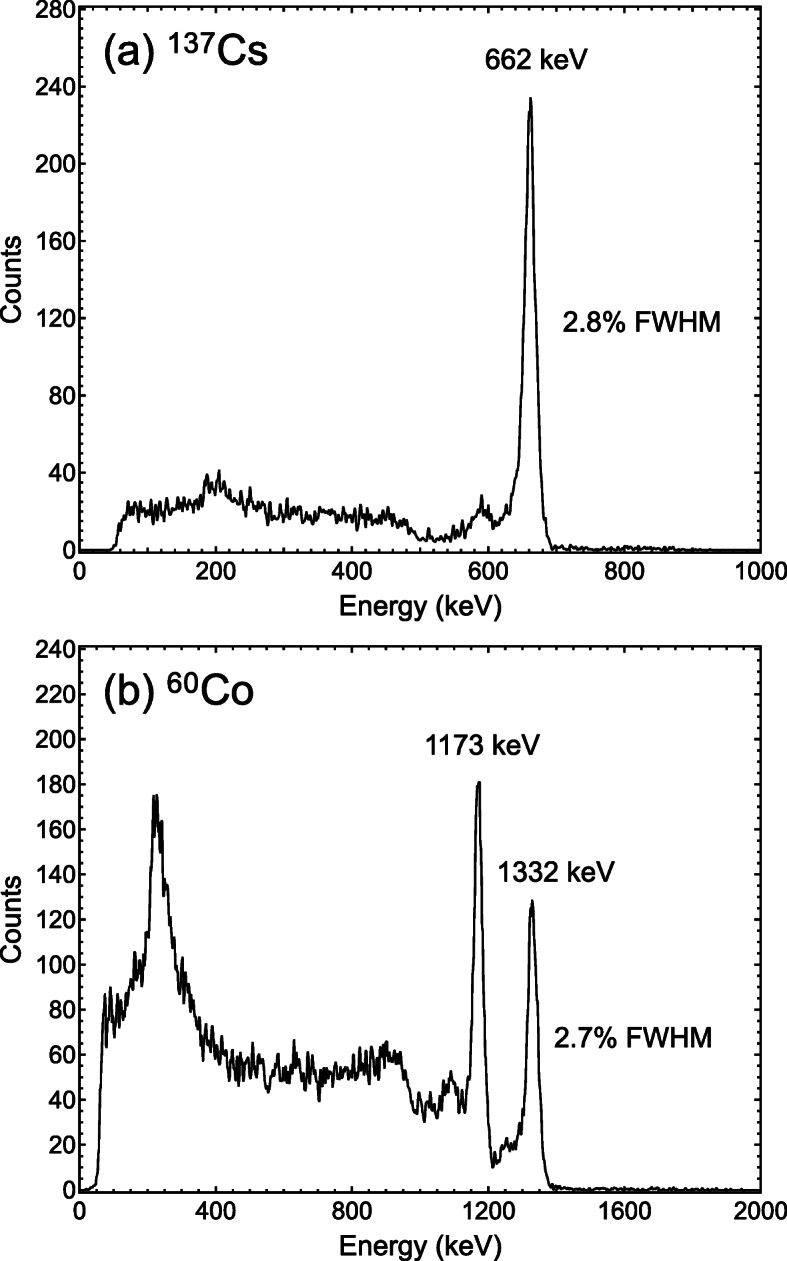
^137^Cs (**a**) and ^60^Co (**b**) gamma-ray spectra obtained from a 1-cm-thick TlBr detector with depth correction and rejection at room temperature

A 1-cm-thick TlBr detector was fabricated from a TlBr crystal grown by the traveling molten zone method in our laboratory. In order to obtain a TlBr crystal with high purity, a commercially available TlBr material was purified by the zone purification method. The size of the detector crystal was approximately 5 mm × 5 mm × 10 mm. The electrodes of the device were constructed by vacuum evaporation of a thallium-based alloy on the crystal. The TlBr detector demonstrated excellent spectroscopic performance at room temperature reflecting the high charge transport prosperities of the crystal and exhibited a high peak-to-Compton ratio originating from the large crystal size and the high atomic number of the material, as shown in Fig. 4.

#### Conclusion

We have succeeded in growing high-quality TlBr crystals for gamma-ray detector fabrication. Because TlBr is extremely adept at stopping gamma-rays, the detectors are applicable to gamma-ray spectroscopy and imaging, especially for nuclear sciences and nuclear medicine. Future studies will be directed toward the implementation of TlBr detectors to practical instruments such as gamma-ray spectrometers and gamma cameras.

## The Physical Society of Japan: 2nd (2021) Fumiko Yonezawa Memorial Prize


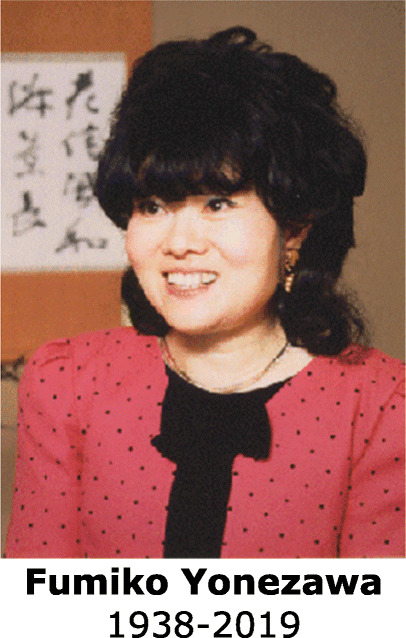


The late Fumiko Yonezawa, emeritus professor of Keio University, made major contributions to physics, such as the development of the coherent potential approximation, and the theory the of metal-insulator transition in liquid selenium. Prof. Yonezawa served as the first female president of the Physical Society of Japan (JPS) and, as the president of the Society for Women Scientists for a Bright Future, she also promoted women scientists.

JPS has established the “Fumiko Yonezawa Memorial Prize” to celebrate the achievements of Prof. Yonezawa and to honor and encourage the activities of the women who are members of JPS.

A few prize winners will be selected once a year, with a maximum of about five receipients. The prize ceremony will be held during the annual meeting of JPS. The prize recipients will give commemorative lectures at JPS meetings within a period of one year after receiving the prize. Winners will receive items such as certificates and honorary shields, as well as additional prizes, namely: (1) paid attendance fees for JPS meetings for the next three years and (2) an exemption of up to 200,000 JPY from (a) publication fees and open access fees for the *Journal of Physical Society of Japan,* and from (b) the article processing charge for the journal, *Progress of Theoretical and Experimental Physics* (valid for 3 years for submissions after the prize is received).

The citations of the winners of the 2nd (2021) Fumiko Yonezawa Memorial Prize are listed below.

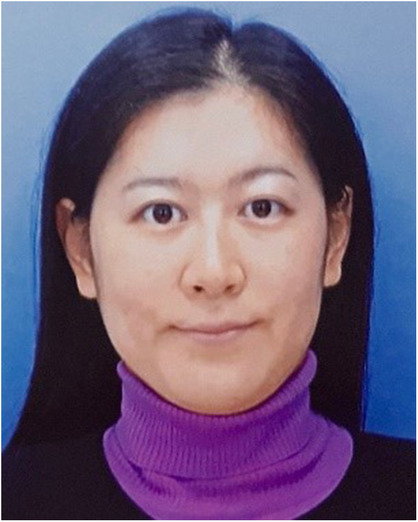



**Yu Nakahama**

**Associate Professor, Kobayashi-Maskawa Institute for the Origin of Particles and the Universe (KMI), Nagoya University**

**Study on new phenomena beyond the standard model of particle physics**

The unification of forces and an understanding of the vacuum are targets of elementary particle physics. Super-symmetric theory (SUSY) has been a strong candidate of the unified theory due to its theoretical simpleness. On the other hand, dark matter has been established from cosmological observations and is commonly thought as yet-unknown elementary particles which SUSY can describe. Discovery of the Higgs particle is a clue to understand the vacuum, and precise investigation of the Higgs field including Higgs self-coupling is thought to be a direction for the next efforts in particle physics.

Verification of the SUSY or beyond the standard particle theory is widely pursued by looking for the production of new particles and/or discrepancies from the standard theory in precise measurements and rare phenomena. Dr. Nakahama introduced a new method to measure CP asymmetries in neutral current decay of B mesons at the Belle experiment and concluded no discrepancy from the standard theory. Then, in the ATLAS (A Toroidal LHC ApparatuS) experiment, she led the search for SUSY gluino and squark inclusively and rejected many SUSY and dark matter models. Her work has had a strong impact in both the field of elementary particle physics and cosmology. She has pioneered the use of deep learning tools in the investigation of the Higgs field. Improvement of the ATLAS trigger performance is indispensable in upgrading the luminosity of the LHC (Large Hardon Collider). Her work as a person in charge of the ATLAS trigger selection project was instrumental in the Run 2 operation of the LHC and was highly praised.

Dr. Nakahama worked at the Belle experiment, at the precision frontier of high energy physics. Then, she continued her research at the ATLAS experiment, at the high energy frontier, and had various achievements. Consequently, she has been promoted to important posts in the group and is a good role model for researchers in particle experiments using an accelerator. She is serving as a committee member for future planning in the high energy physics community. Her contributions continue to propel the field forward. As described above, Dr. Nakahama deserves to receive the Fumiko Yonezawa Memorial Prize as she is a young researcher who is a leader in experimental particle physics.

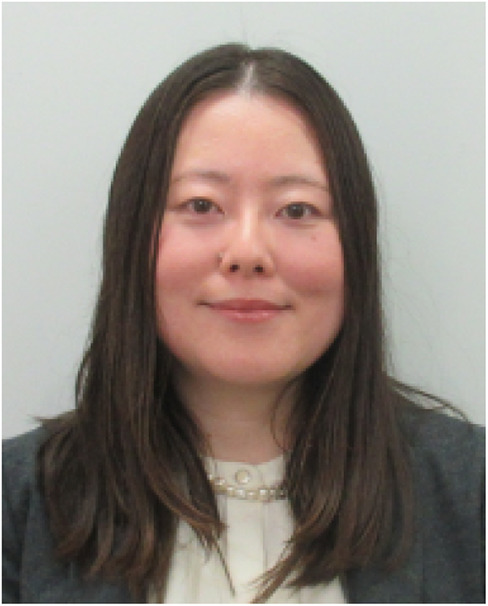



**Emi Minamitani**

**Associate Professor, Institute for Molecular Science**

**Computational study of nanoscale magnetism and phonon**

Dr. Emi Minamitani is a theorist who has been studying magnetism and phonon physics in nanoscale systems by employing cutting-edge computational science approaches. In particular, she uncovered novel quantum phenomena emerging on solid surfaces through close collaborations with experimentalists.

One of her important achievements is the study of the Kondo effect for molecules adsorbed on surfaces. Recently, it has become possible to observe the Kondo effect of atoms/molecules on solid surfaces by STM (Scanning Tunneling Microscope) experiments. Focusing on the flexibility of the shape of molecules as well as their orbital degrees of freedom, she developed a theory incorporating the Kondo effect and the interference effect between different channels in STM currents. Applying this theory, she found the SU(4) Kondo effect with spin/orbital degrees of freedom in a single iron phthalocyanine molecule and further proposed the possibility for reversible control of quantum phase transitions due to competition between the Kondo effect and the magnetic anisotropy. These phenomena were confirmed experimentally.

As another important achievement, she developed a theory of “STM-inelastic electron tunneling spectroscopy (STM-IETS).” In this method, one can detect phonon excitations that are induced by the electron tunneling from an STM tip to a surface. She clarified that the characteristic profile of the tunneling spectrum is determined by the strong momentum/energy dependence of the electron-phonon interaction, thereby explaining the peculiar experimental spectrum observed for the Cu(110) surface. She also succeeded in applying this theory to a graphene/SiC interface. These systematic studies have paved the way to explore phonon excitations on surfaces with the STM-IETS.

Dr. Minamitani’s scientific achievements deserve recognition through the Fumiko Yonezawa Memorial Prize of the Physical Society of Japan.

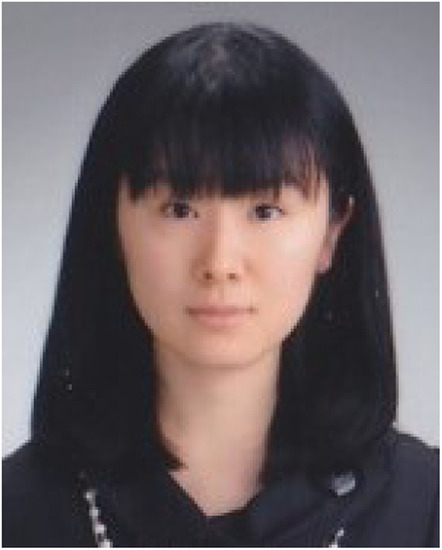



**Hiroko Yokota**

**Associate Professor, Faculty of Science, Chiba University**

**Nano-heterostructures in ferroics and new functionalities appeared at their boundaries**

Dr. Yokota's work has focused on the phase and domain boundaries in ferroic materials. First, for the piezoelectric solid solution system , she has revealed the coexistence of multiple crystal structures by high-resolution neutron diffraction experiments. Moreover, based on the precise analysis of the structurale data with the PDF method, she experimentally confirmed the rotation of polarization, which had been theoretically proposed to cause a giant effect. Second, for inherently non-polar ferroelastic compounds, she used an optical 2nd harmonic generation microscope and successfully observed that the domain boundaries show polarization. These results indicate that the physical properties at domain or heterostructure boundaries are different from bulk properties, which contributes to the establishment of a new field in solid state physics.

The Scope of Dr. Yokota’s research is evident through her many publications, invited talks, and various awards. She is anticipated to be a role model for young women who are researchers and her work deserves to be recognized through the Fumiko Yonezawa Memorial Prize.

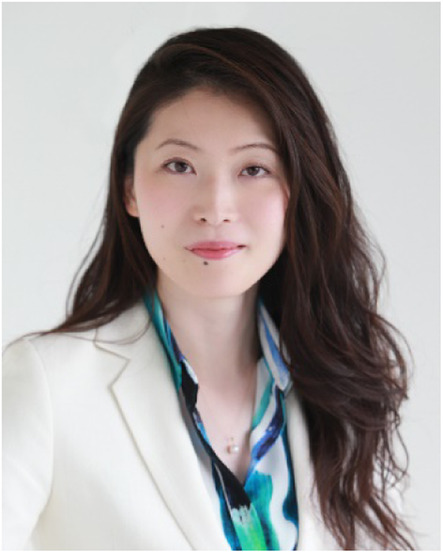



**Hiroko Watanabe**

**Assistant Professor, Research Center for Neutrino Science, Tohoku University**

**Research on cosmic ray variations during grand solar measuring the Earth’s neutrino flux and constraining its composition**

Neutrino detections in Kamioka Mine, which was originally designed to search for proton decay, have achieved significant progress in size and precision since Kamiokande (from 1983), Super-Kamiokande (from 1996), and KamLAND (from 2002). Hyper-Kamiokande is expected to begin in 2027. The renowned results at Kamioka Mine that brought two Nobel prizes to Japan are assoicated to studies in super-nova neutrinos, solar neutrinos, and atmospheric neutrino oscillation. Amongst those results, an especially prominent outcome is from geo-neutrino detections conducted at KamLAND, which is a major achievement of Prof. Hiroko Watanabe. Geo neutrinos are produced by radioactive decays of thorium and uranium in the crust and the mantle of Earth, and are one of the important heat sources of Earth. The heat balance of Earth had been one of the long-standing puzzles in earth science for over two centuries. Prof. Watanabe and her collaborators determined the geo-neutrino flux, and were able to put stringent constraints on heat models of Earth through detailed comparisons between reactor neutrinos and geo-neutrinos. While the first detection of the geo-neutrino by KamLAND was made in 2005, her result established a new interdisciplinary field,- “neutrino earth science,” which integrated neutrino physics and earth science. Prof. Watanabe is the corresponding author of the epoch-mkaing paper titled "Reactor on-off antineutrino measurement with KamLAND" and has presented related works in many invited talks and plenary talks internationally. She is and will continue to be the leading scientist of “neutrino earth science” and she is presently promoting the separation of crust-origin neutrinos and mantle-origin neutrinos with a directionally sensitive neutrino-detection technology. Prof. Watanabe's work deserves to be recognized through the Fumiko Yonezawa Memorial Prize.

## 2020/2021 Annual Meeting of Division of Gravity and Relativistic Astrophysics, Chinese Physical Society

The 2020/2021 Annual Meeting of Division of Gravity and Relativistic Astrophysics of the Chinese Physical Society was successfully held at Northeastern University in Shenyang, China, from April 23 to April 28, 2021. This meeting was organized by Northeastern University and co-organized by Peking University, Beijing Normal University, Chongqing University, Wuhan University, Lanzhou University, and Institute of Theoretical Physics of the Chinese Academy of Sciences. More than 500 researchers participated in this on-site meeting. The number of participants is an exciting piece of news for the whole science community all over the world in face of the pandemic of COVID-19.

**Fig. 5:**
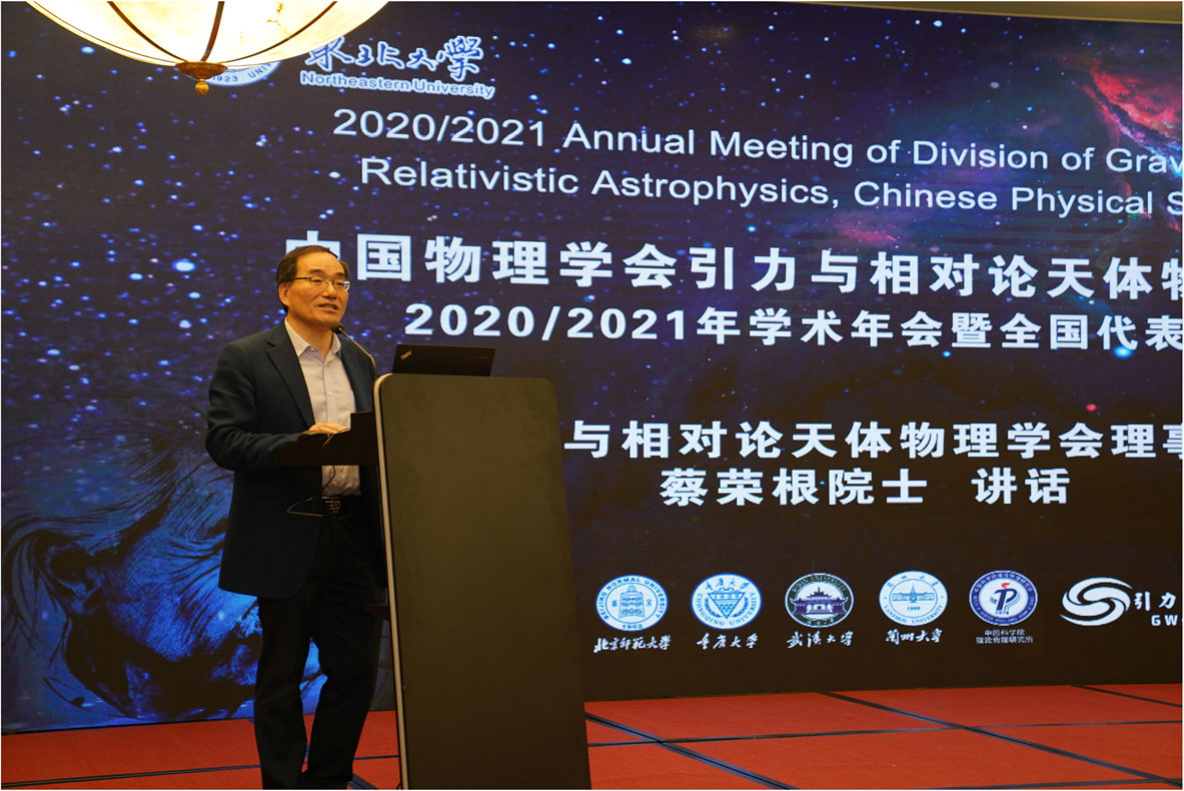
Professor Rong-Gen Cai gave a passionate speech at the opening ceremony of the meeting

Professor Rong-Gen Cai (Academician of the Chinese Academy of Sciences and chair of the committee of the division) gave a passionate speech at the opening ceremony of the meeting (Fig. 5). Professor Cai said that the world is experiencing a phenomenal change that has not been seen in the past century. Global scientific and technological innovation has experienced an unprecedented dense and active period. In this historical period, our country, China, remarked Cai, is paying particular attention to the investment in basic research and the development of top talents in science and technology more than ever before. Basic research is the source of the entire scientific system, and the research of gravity and astrophysics is one of the most fundamental parts of basic research. In recent years, major breakthroughs have frequently appeared in the field of gravity and astrophysics. For instance, the detection of gravitational waves and the capture of the first image of a black hole have opened new windows and provided new tools for exploring various mysteries of the universe in greater depth. He added: “In the past four years, three Nobel Prizes in Physics have been awarded to the field of gravity and astrophysics, a recognition that adequately shows that the research in our field is embarking on a golden age.”

With the construction of a number of major scientific facilities, such as the FAST telescope (“China’s Sky Eye”), China will definitely see an explosion of important achievements in this scientific field. Cai also noted that “at present, our country (China) is undergoing a comprehensive reform of the science and technology system so as to enhance the effectiveness of the innovation system and stimulate greater innovation vitality.” Professor Cai called on young people to keep in step with the times, seize any opportunities that appear, unswervingly devote themselves to the frontiers of science, and strive for major innovations with global influence. He noted that there is a need to create a good innovation environment for young talents and accelerate the formation of a training and competition mechanism conducive for the growth and emergence of talents. He reiterated that it is important to cultivate and bring up a large number of leading experts in China in the field of gravity and astrophysics.

There were eleven plenary speakers at the meeting: Xuelei Chen (National Astronomical Observatories, Chinese Academy of Sciences/Northeastern University), Hong Lv (Tianjin University), Yawen Sun (University of Chinese Academy of Sciences), Dechang Dai (Yangzhou University), Xilong Fan (Wuhan University), Wen Zhao (University of Science and Technology of China), Feng Yuan (Shanghai Astronomical Observatory, Chinese Academy of Sciences), Puxun Wu (Hunan Normal University), Andre Costa (Yangzhou University), Yefei Yuan (University of Science and Technology of China), and Di Li (National Astronomical Observatories, Chinese Academy of Sciences). There were also five parallel conference themes at the meeting, namely, Gravitational Theory I, Gravitational Theory II, Gravitational Wave Physics, Cosmology and Astrophysics, and Black Hole Physics, and a total of 169 oral presentations. Participants at the meeting enjoyed an in-depth and extensive exchange of the latest research results and updates in the field of gravity and astrophysics, covering the frontier issues in gravitational theory, gravitational wave physics, black hole physics, quantum gravity, gravitational experiments, quantum field theory in curved spacetime, nuclear astrophysics, dark matter and dark energy, early universe, and cosmological probes.

This meeting also witnessed the general election of the Division of Gravity and Relativistic Astrophysics of the Chinese Physical Society. On April 24, the eleventh committee was elected, and the first plenary session of the new committee was held on the same evening to elect the members of the standing committee. Rong-Gen Cai was elected as the chair of the committee; Yi Ling, Bin Wang, Hongwei Yu, and Xin Zhang were elected as the vice-chairs of the committee. Yu-Xiao Liu was appointed as the secretary general, and Zhou-Jian Cao was appointed as the vice-secretary general. The election result has been submitted to the standing council of the Chinese Physical Society for consideration and approval.

**Fig. 6:**
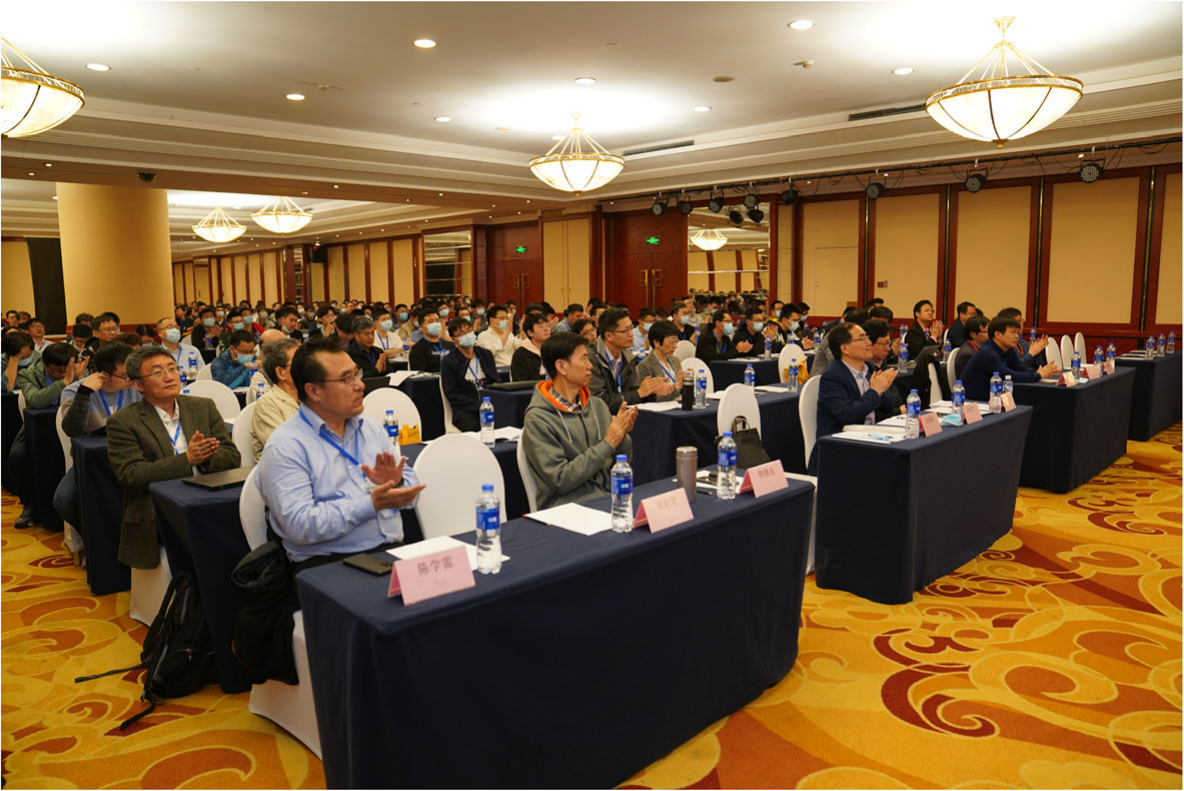
Photo taken in the main conference venue

**Fig. 7:**
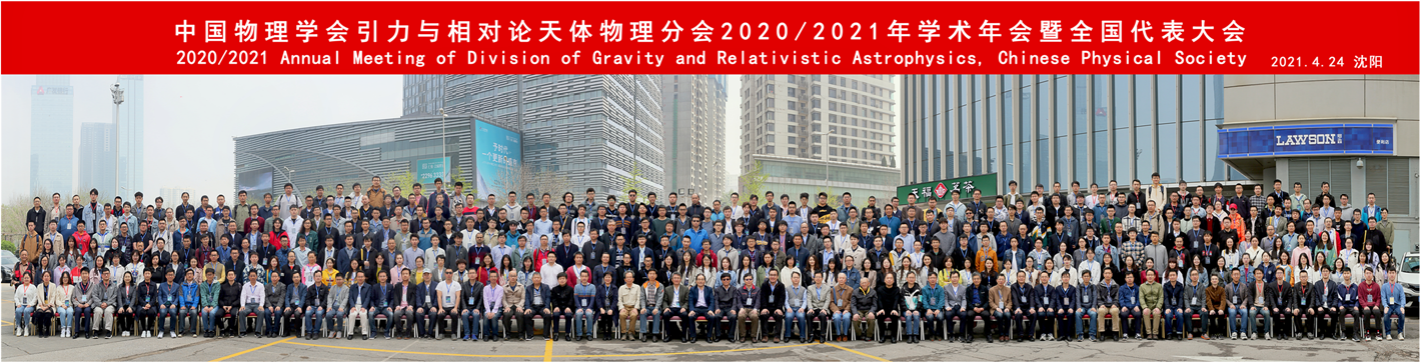
Meeting group photo

This meeting had also attracted a large number of top researchers in China in the field of gravity and astrophysics. Many researchers had good academic exchanges of the latest research results from different groups and updated themselves with the progress in the fields of gravity, cosmology, astronomy, and astrophysics. Ironically, even with the pandemic, the scale of the meeting is the largest ever organized. The high level of the presentations and the extensive discussions have played a significant role in the promotion of exchanges, cooperation, and development in these fields. Since its inception in 1974, the Annual Meeting of Division of Gravity and Relativistic Astrophysics, Chinese Physical Society, has been going strong for the last 42 years, and it is now one of the most prestigious academic events in the physics community of China (Figs. 6 and 7).
